# Supplementary material for: Observation of topological superconductivity in a stoichiometric transition metal dichalcogenide 2M-WS2
Source: Nat Commun. 2021 May 17;12:2874. doi: 10.1038/s41467-021-23076-1 (PMC8129086; doi:10.1038/s41467-021-23076-1)
Supplement: Supplementary file 1 — Supplementary Information [file 41467_2021_23076_MOESM1_ESM.pdf]

Supplementary Information for

Observation of Topological Superconductivity in a Stoichiometric Transition Metal Dichalcogenide 2M-WS_2

Li *et al.*

This file includes

Supplementary Note 1 Crystal structure of transition metal dichalcogenides

Supplementary Note 2 Characterization: X-ray diffraction and core-level spectroscopy

Supplementary Note 3 *Ab initio* calculation on the charge density from the topmost S-atom layer

Supplementary Note 4 In-plane electronic structures

Supplementary Note 5 k_z dependence of the bulk electronic states

Supplementary Note 6 Details of resolved surface and bulk bands near the surface Dirac point

Supplementary Note 7 Extraction of the superconducting gap by BCS spectral function

Supplementary Note 8 Anisotropy of the surface Dirac cone

Supplementary Note 9 Temperature evolution of surface and bulk bands

Supplementary Note 10 Bogoliubov quasiparticle

Supplementary Figures 1-10

Supplementary Note 1: Crystal structure of transition metal dichalcogenides

Transition metal dichalcogenides (TMDs) are typically labeled as MX_2, where M stands for a transition metal element (e.g. W) and X stands for a chalcogen element (e.g. S). Usually, they have two types of building blocks, commonly known as the H-type and T-type. H- and T-types of building blocks and their monolayer structures are depicted in Supplementary Figs. 1a and 1b, respectively. Both types of building blocks have the chalcogen-metal-chalcogen sandwich structure. In the H-type structure, the transition metals are trigonal prismatic coordinated by chalcogen atoms, whereas in the T-type structure, the coordination is octahedral. In some TMDs, such as WTe_2 and $MoTe_2$, the T-type structure is not thermally stable, and it undergoes 2×1 Peierls distortion at low temperature. The T-type structure is then transformed into the T'-type structure as illustrated in Supplementary Fig. 1c.

These three types of building blocks (H, T and T') form the basic structures of a monolayer of TMDs. The variation in the stacking geometry of neighboring layers further increases the diversity of bulk crystal structures. Supplementary Figs. 1d-f presents several examples. As shown in Supplementary Figs. 1e and 1f, although T' and 2M phases have the same monolayer structure, the monolayers of the T' phase stack through a glide mirror operation, whereas in the 2M phase, they stack through a translation operation. The translational vectors are indicated as magenta arrows in Supplementary Figs. 1f and 1g. The detailed crystallography information is listed in Supplementary Fig. 1h.

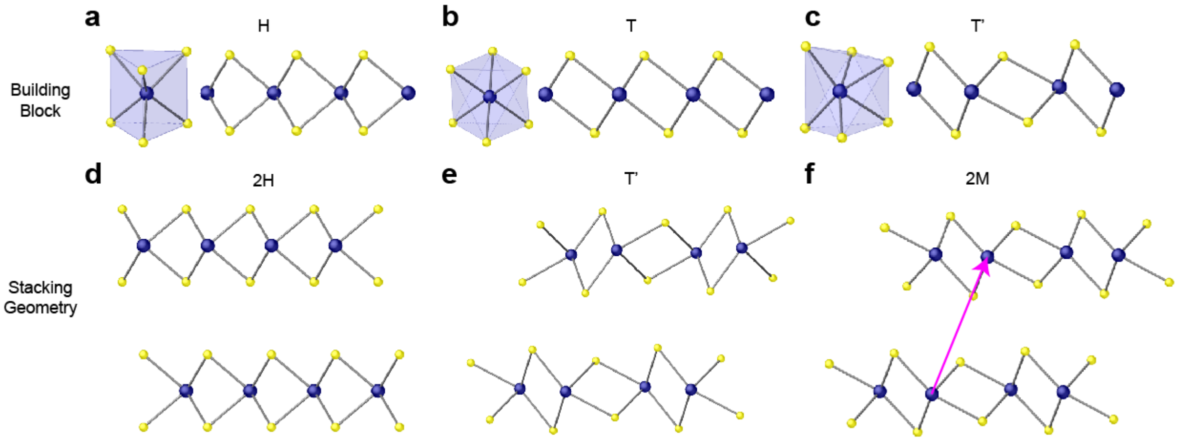

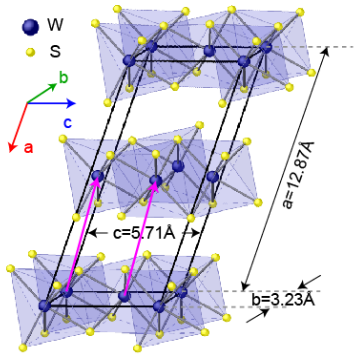

h

Overview	Site	Occ.	x/a	y/b	z/c
Chemical Formula: WS_2	S1	1	1.1030	1.0000	0.4741
Z: 4	S2	1	0.3842	1.0000	0.1187
Space Group: C2/m (12)	S3	1	0.6030	0.5000	0.4741
Crystal System: Base Centred Monoclinic	S4	1	0.8863	0.4983	0.1207
a: 12.87 Å	S5	1	0.8486	1.0041	0.5906
b: 3.23 Å	S6	1	0.6388	1.0000	0.0091
c:5.71 Å	S7	1	0.3484	0.5000	0.5838
α: 90°	S8	1	0.1409	0.4983	0.0111
β: 112.9°	W1	1	0.9873	0.5000	0.5928
γ: 90°	W2	1	0.4999	0.5000	0.0000
Cell Volume: 218.436 $Å^3$	W3	1	0.4873	1.0000	0.5928
Density: 7.54 g/cm^3	W4	1	0.9999	1.0000	0.0000

Supplementary Figure 1 | Crystal structure of transition metal dichalcogenides. (**a-c**), The building blocks (left panel) and side views (right panel) of monolayer H, T and T' phases. (**d-e**), Stacking geometry of 2H, T' and 2M phases. (**g**), 3D crystal schematic of 2M-WS2. The translational vectors between neighbouring layers are indicated by the magenta arrows in (**f**) and (**g**). (**h**), Crystallography information of 2M-WS2.

Supplementary Note 2: Characterization: X-ray diffraction and core-level spectroscopy

The crystal structure of 2M-WS2 is investigated by both single-crystal and powder X-ray diffraction (XRD). The single-crystal XRD (Supplementary Fig. 2a) was performed using Mo Kα radiation by the Rigaku Oxford Diffraction at the Department of Physics, University of Oxford. The beam spot size is 10-200 microns in diameter. The data was collected and analyzed by the CrysAlisPro software, as shown in the upper row of Supplementary Fig. 2a. The experimentally measured lattice constant is $a = 12.95\text{Å}, b = 3.25\text{Å}, c = 5.79\text{Å}, \alpha = 90°, \beta = 112.9°, \gamma = 90°$ in the base-centred monoclinic space group $C_{2/m}$ (No.12), consistent with previous study and calculations[1,2] ($a = 12.87\text{Å}, b = 3.23\text{Å}, c = 5.71\text{Å}, \alpha = 90°, \beta = 112.9°, \gamma = 90°$). The corresponding single-crystal XRD pattern simulation (lower row of Supplementary Fig. 2a) was performed using the SingleCrystal software of the Crystalmaker Suite. The experimental and simulated single-crystal XRD show good agreement.

The powder XRD (Supplementary Fig. 2b) was performed on a Bruker D8 QUEST diffractometer equipped with Mo Kα radiation. The index of the diffraction peaks was calculated by the CrystalDiffract 6 software of the Crystalmaker Suite.

The core-level spectroscopy (Supplementary Fig. 2c) was conducted at I05 high-resolution ARPES branch of Diamond Light Source (UK), using a photon energy of 74 eV. The W 5p and 5f core-level peaks are observed. The inset of Supplementary Fig. 2c is the image of the as-grown crystal surface. The perpendicular textures on the crystal surface are parallel to crystallographic directions b and c, respectively, which can be a simple guide for sample alignment in the experiment.

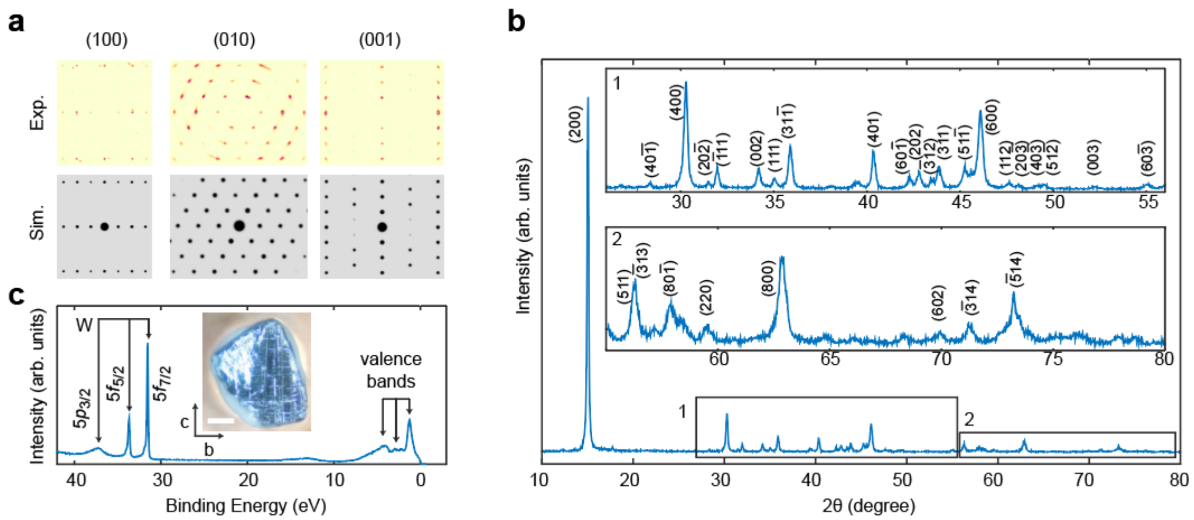

Supplementary Figure 2 | Basic characterization: X-ray diffraction and core-level spectroscopy. (**a**), Comparison of experimental (upper row) and simulated (lower row) single-crystal XRD pattern along (100), (010) and (001) direction. (**b**), Indexed powder XRD pattern of 2M-WS_2. (**c**), Core-level spectroscopy of 2M-WS_2. The inset shows the optic image of the crystal. The scale bar is 0.5 mm.

Supplementary Note 3: *Ab initio* calculation on the charge density from the topmost S-atom layer

Ab initio calculation on the charge density distribution in the bilayer 2M-WS_2 is detailed in Supplementary Fig. 3. The energy range is selected to be 300~350 meV above the Fermi level, which corresponds to the bias voltage of +340 mV applied in the STM measurement. The side view of the charge density (Supplementary Fig. 3b) can be easily matched with the crystal structure in Supplementary Fig. 3a. The charge distribution on the topmost S atom layer (Supplementary Fig. 3c) and middle W atom layer (Supplementary Fig. 3d) is extracted. As illustrated in Fig. 1e in the main text, the STM topography shows a consistent pattern with the charge density distribution on the topmost S atom layer, which indicates a defect-free S-termination.

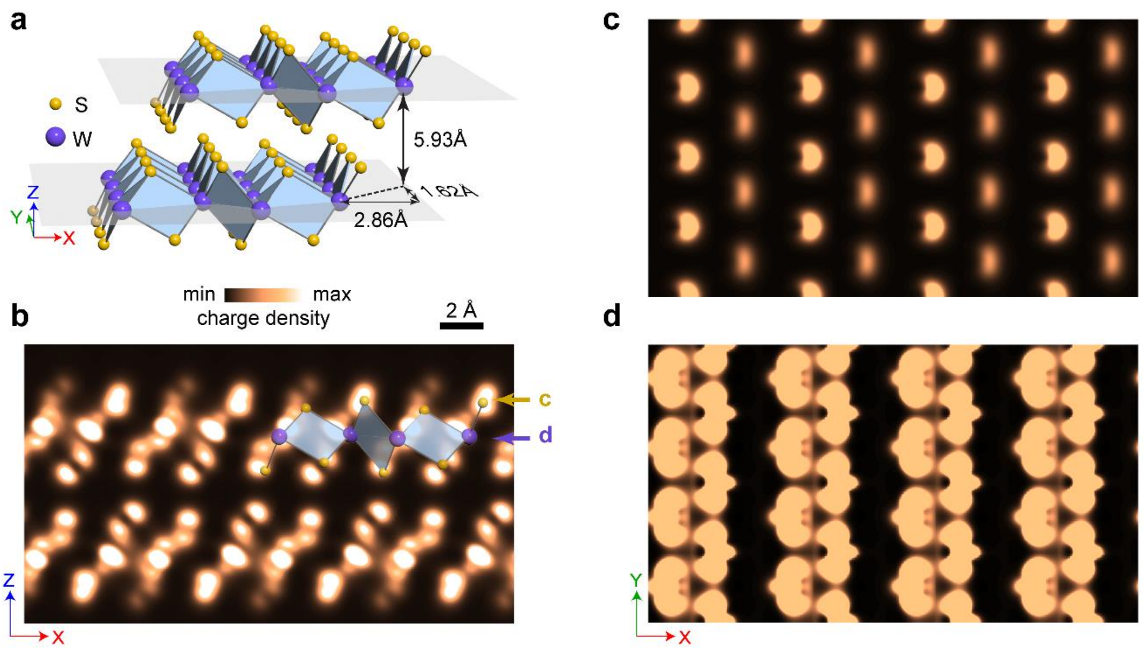

Supplementary Figure 3 | *Ab initio* calculation on the charge density distribution. (**a**), Illustration of the 2M-WS_2 crystal structure. (**b**), Side view of charge density distribution with the side view of the crystal structure overlaid. (**c**), Charge density distribution on the topmost S-atom layer. (**d**), Charge density distribution on the middle W-atom layer.

Supplementary Note 4: In-plane electronic structures

Supplementary Fig. 4 presents a more detailed comparison between ARPES measurements and *ab initio* calculation on the in-plane electronic structures of 2M-WS_2. One can see the quasi-one-dimensional Fermi surface along $\bar{\Gamma}\bar{X}$ from the broad Fermi surface mapping shown in Supplementary Fig. 4a, carried out at Spectromicroscopy branch of Elettra synchrotron using a photon energy of 74 eV for large momentum space coverage. Details of comparison of band dispersions and energy contours between the experiment and the calculation in Supplementary Figs. 4b and 4c show good agreement. The band dispersions and zoomed-in energy contours are measured at Spectromicroscopy branch of Elettra synchrotron using a photon energy of 27 eV for better energy and momentum resolution. The *ab intio* calculations show the (100) projection of the local density of states (LDOS) of the bulk electronic states.

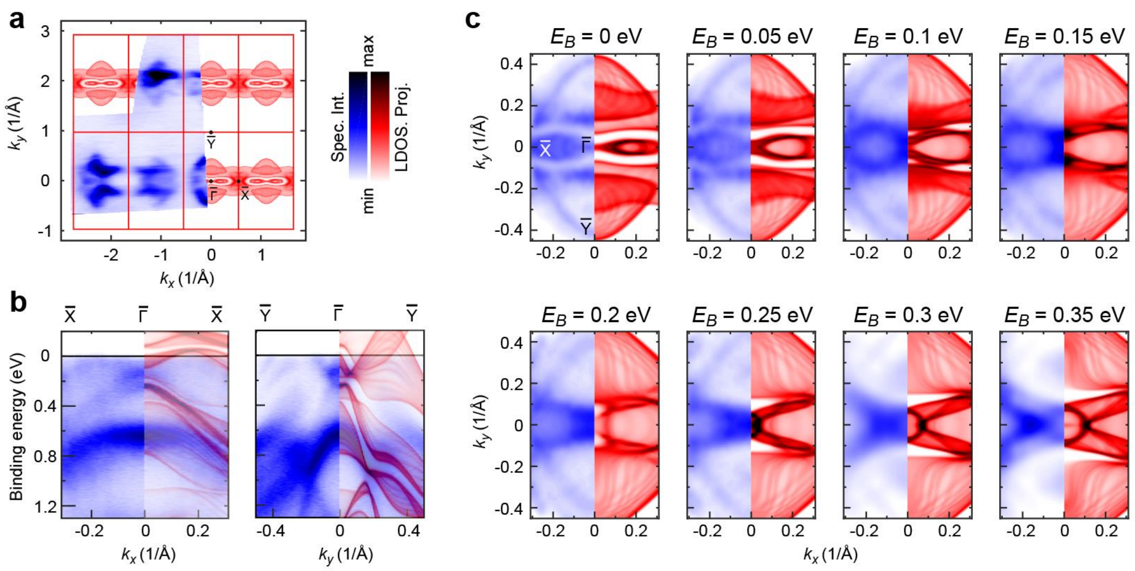

Supplementary Figure 4 | In-plane electronic structures. (**a**), Comparison of Fermi surfaces between the broad ARPES mapping and the corresponding *ab initio* calculations. (**b**), Comparison between the ARPES measured band dispersions and the corresponding *ab initio* calculations along $\bar{X} - \bar{\Gamma} - \bar{X}$ left panel) and $\bar{Y} - \bar{\Gamma} - \bar{Y}$ (right panel), respectively. (**c**), Comparison between the ARPES measured energy contours and the corresponding *ab initio* calculations. The experimental plots in (**c**) are symmetrized with respect to $k_y = 0$ according to the crystal symmetry.

Supplementary Note 5: k_z dependence of the bulk electronic states

During the photoemission process, the in-plane momentum ($k_{\parallel}$) of electrons in the sample is obtained directly from the photoelectrons' inplane momentum component due to the momentum conservation. However, the out of plane momentum perpendicular to the crystal surface (k_z) is not conserved due to the surface electric field. Under the free-electron final state approximation and use a potential parameter V_0 (also known as the inner potential), we can derive the k_z as:

$$k_z = \frac{\sqrt{2m_e(E_k \cos^2 \theta + V_0\ )}}{\hbar}$$

where θ is the emission angle, m_e is the effective electron mass and E_k is the kinetic energy of the photoelectron, which satisfies:

$$E_k = h\nu - w - E_B$$

where $h\nu$ is the photon energy, w is the work function and E_B is the electron binding energy.

As V_0 is a material-dependent parameter, we typically perform photon energy dependent ARPES measurement to cover enough k_z range (ideally more than one Brillouin zone). By comparison with corresponding calculation, we can estimate the k_z value of the ARPES spectrum under specific photon energy[3].

For 2M-WS_2, from the photon energy dependent measurement ($h\nu = 30{\sim}90\ eV$), we estimate $m_e = 1.15m_0$ and $V_0 = 6.4\ eV$. Supplementary Fig. 5 presents the comparison between ARPES measurements and *ab initio* calculation on the k_z dependence of the bulk electronic states. On the $k_y - k_z$ plane, the Fermi surfaces consist of small electron pockets near the Γ plane (indicated by the red curves α), large hole pockets centred at Z plane (indicated by the orange curves β_2) and a small hole pocket along ΓZ (indicated by the orange curves β_1). In the energy range from the Fermi level to binding energy of 0.4 eV, there are three bands (α, β and γ) and two of them (α and β) cross the Fermi level, as marked in Supplementary Figs. 5b and 5d. The ARPES measurement results are in general consistent with the *ab initio* calculations.

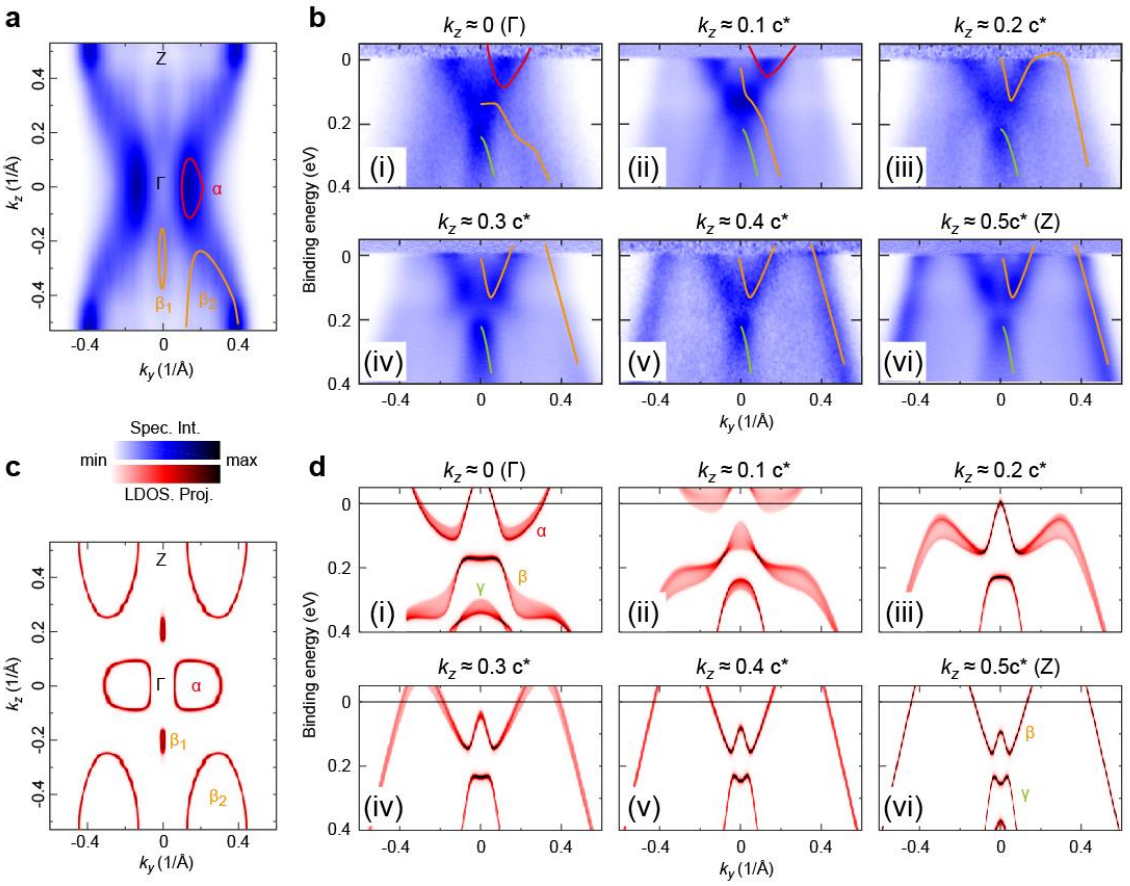

Supplementary Figure 5 | k_z dependence of the bulk electronic states. (**a**), ARPES measured Fermi surfaces on the $k_y - k_z$ plane. (**b**), ARPES measured k_z dependence of band dispersions from the Γ plane ($k_z = 0$) to the Z plane ($k_z = 0.5a^*, where\ a^* = 2\pi/a$, and a is the out-of-plane lattice constant). (**c**) and (**d**), Corresponding *ab initio* calculation to (**a**) and (**b**). Band α, β and γ are indicated as red, orange and green curves, respectively, in (**a**) and (**b**). Abbreviation, LDOS: local density of states. The experimental plots are symmetrized with respect to $k_y = 0$ according to the crystal symmetry.

Supplementary Note 6: Details of resolved surface and bulk bands near the surface Dirac point

Since the surface Dirac point is slightly above the Fermi level ($E - E_F \approx 10\, meV$), we managed to access the upper Dirac cone via surface potassium doping, as shown in Supplementary Figs. 4b and 4e. Although the spectra are broadened due to the surface disorders introduced by this process, the surface and bulk bands near the surface Dirac point can be resolved by careful analysis of the momentum distribution curves (MDCs), as shown in Supplementary Fig. 6. The MDCs extracted along $\bar{Y} - \bar{\Gamma} - \bar{Y}$ in the momentum range (-0.1Å,0.1 Å) can be fitted with four Lorentzian peaks above the surface Dirac point and three at the Dirac point, as shown in Supplementary Figs. 6d-f, consistent with the *ab initio* calculation.

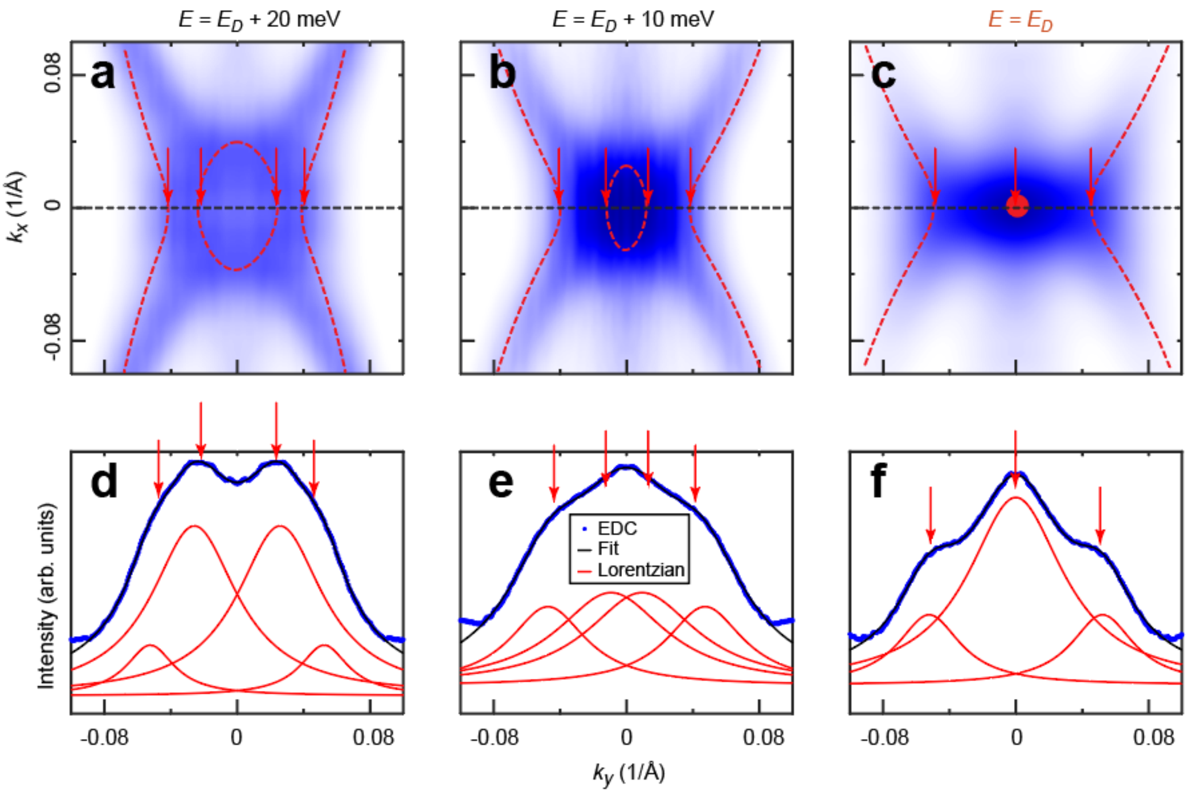

Supplementary Figure 6 | Details of resolved surface and bulk bands near the surface Dirac point. (**a-c**), Energy contours at three different energies near the surface Dirac point. (**d-f**), MDCs (blue dotted lines) extracted along the dashed lines from (**a-c**), fitted with multiple Lorentzian peaks (red solid lines). The Fermi surface mapping is symmetrized with respect to $k_y = 0$ and $k_x = 0$ according to the crystal symmetry.

Supplementary Note 7: Extraction of the superconducting gap by the spectral function

Superconducting gap magnitude was quantitatively determined by the spectral function at k_F

$$A(k_F, E) = A_0 \frac{\Sigma_2}{(E - \Sigma_1)^2 + \Sigma_2^2}.$$

Σ_1 and Σ_2 are the real and imaginary part of the self energy Σ, which has the form[4]:

$$\Sigma(k_F, E) = -i\Gamma + \frac{\Delta^2}{E + i\gamma}.$$

Here Γ is a single-particle scattering rate (here we assume it as energy-independent for simplicity). The second term is the BCS self-energy (corresponding to the diagonal term of the Nambu-Gorkov propagator), where Δ is the superconducting gap and gamma is a small real positive quantity to avoid the divergence when $E = 0$.

For the EDCs extracted at the Fermi momentum k_F, the spectral intensity can be described as the ARPES intensity $I(E) = [A(k_F, E) f(E; T)] * R(E; \Delta E)$, where $A(k_F, E)$ is the spectral function shown above, $f(E; T)$ is the Fermi-Dirac distribution function and $R(E; \Delta E)$ is the convoluted energy resolution function. Since $A(k_F, -E) = A(k_F, E)$ and $f(-E) = 1 - f(E)$, one can obtain that the symmetrized EDCs as $I(E) + I(-E) = A(k_F, E) * R(\omega; \Delta E)$.

Supplementary Fig. 7a presents the temperature evolution of the deconvoluted ARPES EDCs, which are obtained by I_{deconv} = deconvlucy (I, R). R is the energy resolution function – Gaussian function (normal distribution) with standard deviation σ = 0.9 meV, extracted from the Au spectra (see Supplementary Fig. 7b). The deconvlucy algorithm is based on maximizing the likelihood that the deconvoluted data I_{deconv} is an instance of the original data I under Poisson statistics. The deconvolution is performed using the built-in function *deconvlucy* in MATLAB. The fitted temperature-dependent superconducting gap Δ exhibits a rapid decrease to zero whereas the scattering rate Γ shows significant enhancement near the superconducting transition temperature T_C, as shown in Supplementary Fig. 7c.

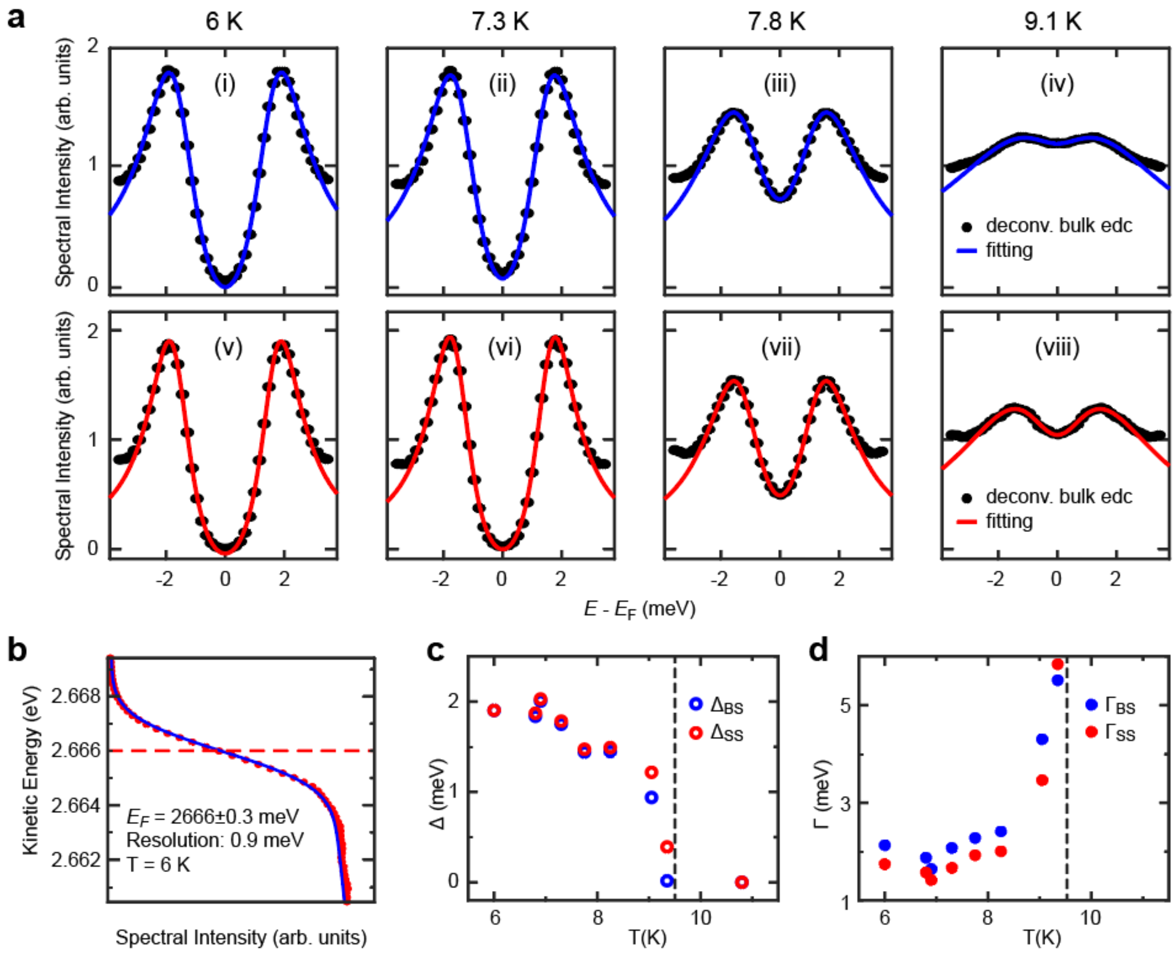

Supplementary Figure 7 | Extraction of superconducting gap magnitude. (**a**), Deconvoluted EDCs of the bulk state (i)-(iv) and the surface state (v)-(viii) fitted by the minimal model (see text). (**b**), Fermi energy determined by Au spectra. The Fermi-Dirac function fitting shows that the Fermi level of the laser-ARPES measurements is 2.666 meV and the energy resolution is 0.9 meV. The Au spectra are collected at T = 6 K. (**c, d**), Temperature-dependent superconducting gap Δ and the scattering rate Γ.

Supplementary Note 8: Anisotropy of the surface Dirac fermion

The surface Dirac fermion in 2M-WS_2 is highly anisotropic, as indicated in Figs. 3e-f in the main text. In Supplementary Fig. 8, we can investigate the anisotropy of the TSS by extracting the Fermi momenta at different binding energies near the Dirac point along two perpendicular directions, $\bar{Y} - \bar{\Gamma} - \bar{Y}$ and $\bar{X} - \bar{\Gamma} - \bar{X}$, as shown in Supplementary Fig. 8c. Since the band dispersion near the Dirac point is approximately linear, the anisotropy of the Fermi velocity can be estimated as $\frac{v_y}{v_x} \approx \frac{\Delta k_x}{\Delta k_y} = 1.8 \sim 3.5$.

This result is consistent with a recent STM study[2]. Theoretically, the Majorana bound state extends in real space with a size of $\sim \hbar v_F / \Delta$ (the same scale as the Ginzburg-Landau coherence length, which can be extracted from the STM experiment). The ratio of the coherence lengths of Majorana bound states along two perpendicular directions is $\frac{\xi_{s,c}}{\xi_{s,b}} = 3.26$, estimated by the STM experiment. If one assumes that the superconducting gap Δ is nearly isotropic, as shown in Fig. 4g in the main text, then the anisotropy in the real space (coherence length) is comparable with one in the reciprocal space (Fermi momentum).

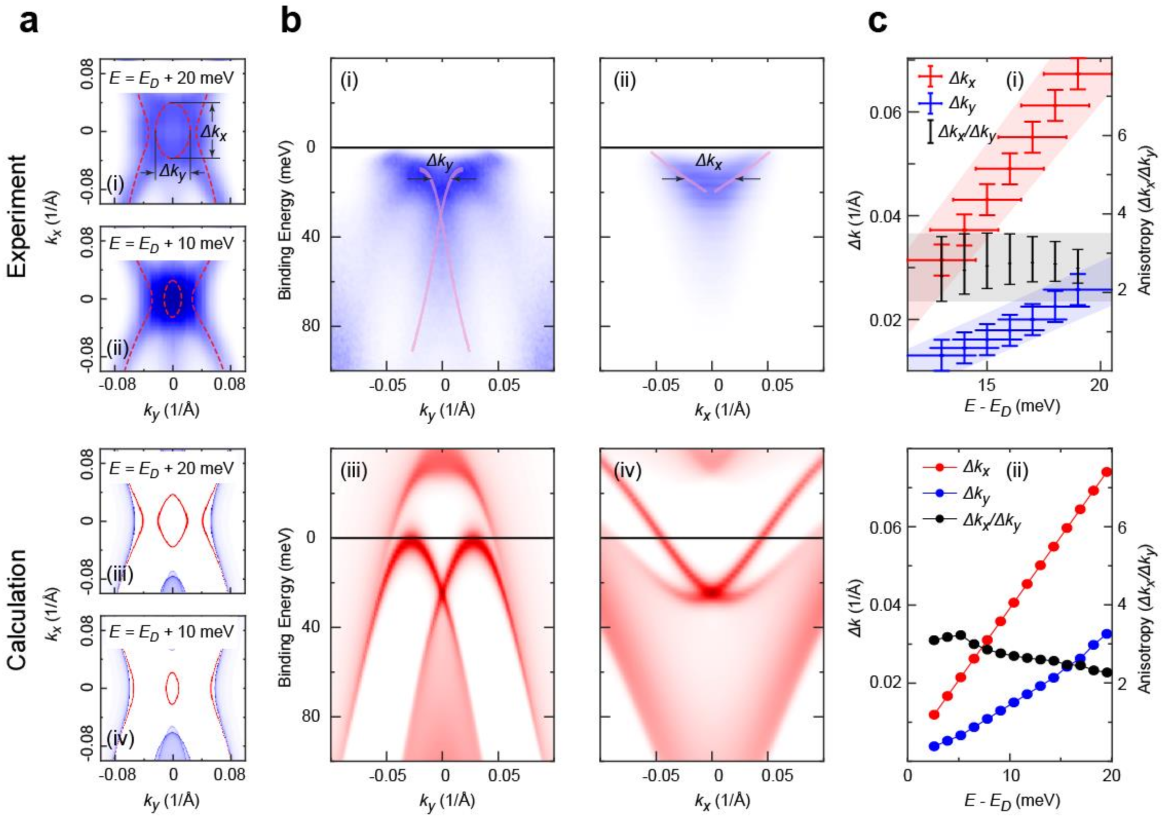

Supplementary Figure 8 | Anisotropy of the surface Dirac cone. (**a**), Comparison between ARPES (i and ii) and *ab initio* calculation (iii and iv) on the anisotropic Fermi surface near the Dirac point. (**b**), Comparison between ARPES (i, $\bar{Y}-\bar{\Gamma}-\bar{Y}$; ii, $\bar{X}-\bar{\Gamma}-\bar{X}$) and *ab initio* calculation (iii, $\bar{Y}-\bar{\Gamma}-\bar{Y}$; iv, $\bar{X}-\bar{\Gamma}-\bar{X}$) on the band dispersion. (**c**), Comparison between the anisotropic Fermi momenta extracted from ARPES (i) and *ab initio* calculation (ii). The experimental plots are symmetrized with respect to $k_y = 0$ and $k_x = 0$ according to the crystal symmetry. Error bar represents the confidence interval of the fitting under 95% confidence level.

Supplementary Note 9: Temperature evolution of surface and bulk bands

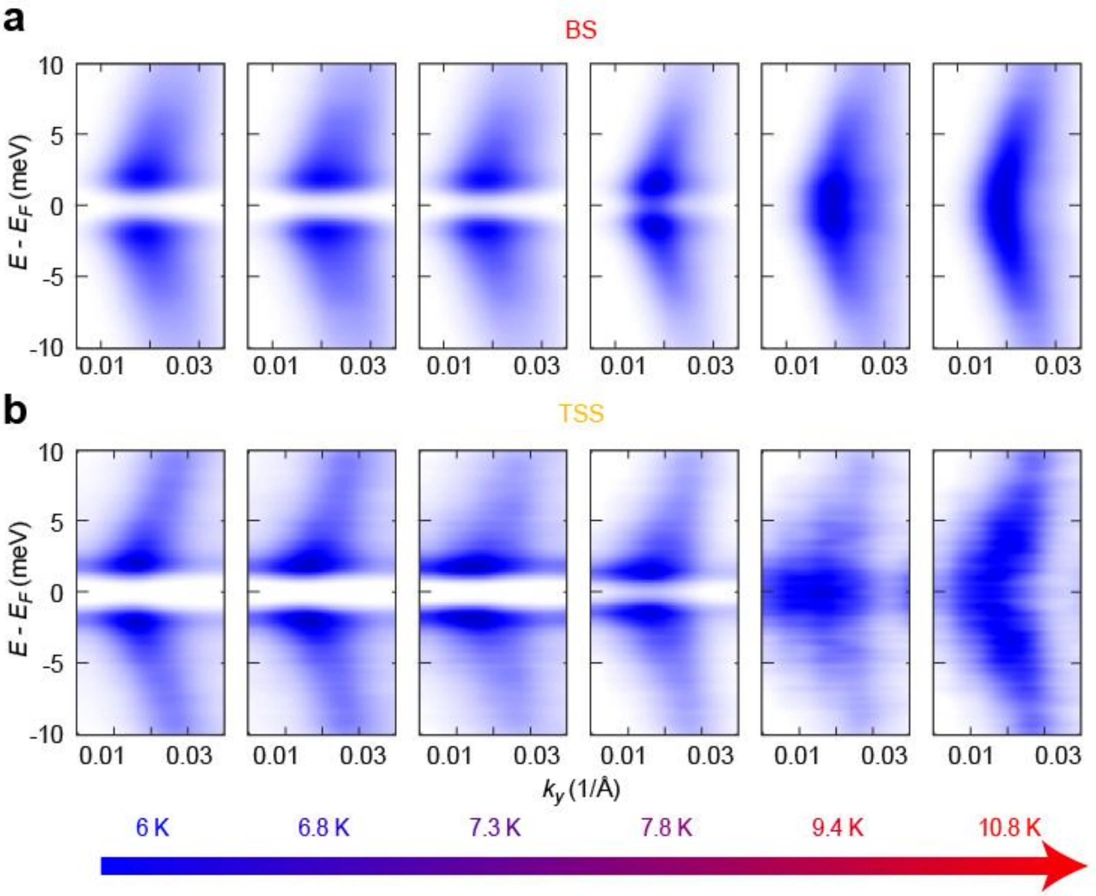

Supplementary Figure 9 | Temperature dependence of the superconducting gap on the bulk state (BS) (a) and the topological surface state (TSS) (b).

Supplementary Note 10: Bogoliubov quasiparticle

ARPES spectrum represents the occupied part of the single-particle spectral function, which is modified near the Fermi level due to the Fermi-Dirac function cut-off. An approximate way to remove this effect is to divide ARPES intensity by an effective Fermi-Dirac function. This is generated from the convolution of the Fermi-Dirac function at the measured temperature with the Gaussian energy resolution function. This standard procedure[5] allows us to trace the Bogoliubov quasiparticle dispersion above E_F, where thermal population leads to appreciable spectral weight at finite temperature, as shown in Supplementary Fig. 10.

Please note that this is an approximated method due to the incommutability between multiplication (Fermi-Dirac function) and convolution (Gaussian resolution function). This approximation works more accurate when the temperature broadening is significantly larger than energy resolution ($kT \gg \Delta E$, e.g., high-T_c superconductor scenario[5]). However, for the ARPES experiment on 2M-WS_2, energy resolution and temperature broadening are comparable ($\Delta E = 0.9$ meV, $k_B T_c = 0.8$ meV). The inaccuracy of this method might result in the intensity and energy asymmetry of upper and lower Bogoliubov quasiparticles shown in Supplementary Fig. 10.

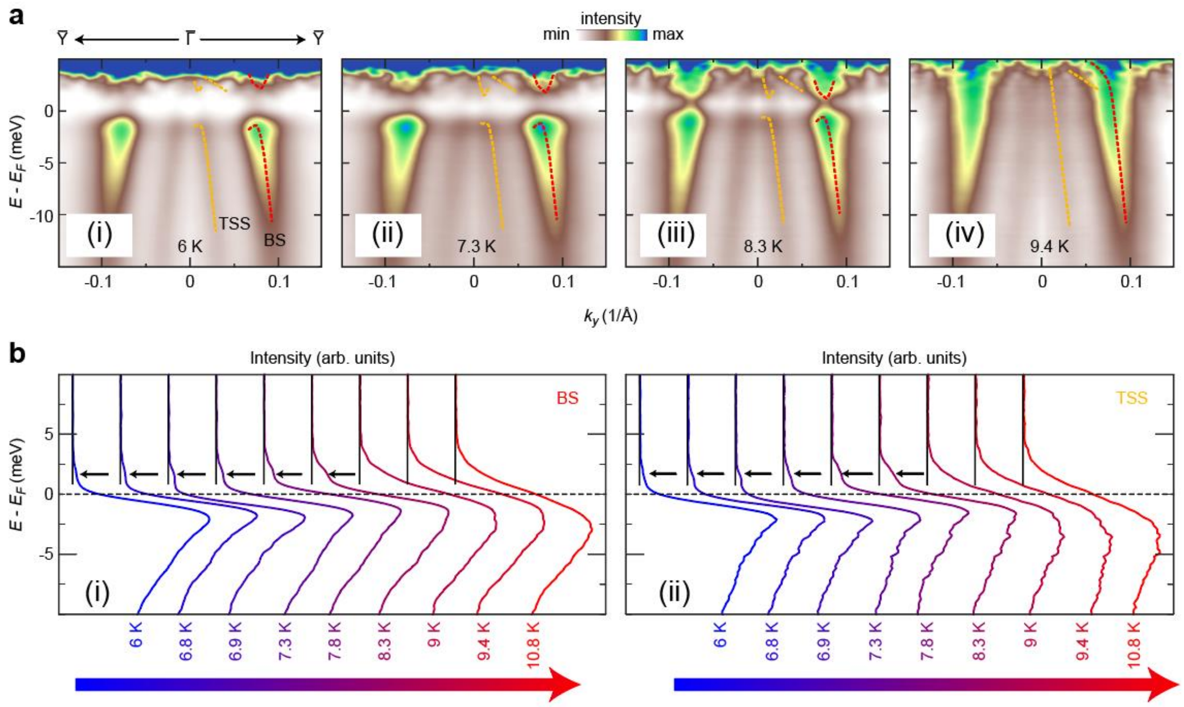

Supplementary Figure 10 | Bogoliubov quasiparticle. The topological surface states (TSS) and bulk states (BS) are indicated by the orange and red dashed lines, respectively. (**a**), Image plots of Fermi-Dirac-function divided ARPES spectrum along $\bar{Y} - \bar{\Gamma} - \bar{Y}$ direction at four different temperatures. The topological surface states (TSS) and bulk states (BS) are indicated by the orange and red dashed lines, respectively. (**b**), Temperature evolution of the raw EDCs extracted at the Fermi momentum k_F of the bulk state (BS) (i) and the topological surface state (TSS) (ii). The experimental plots are symmetrized with respect to $k_y = 0$ according to the crystal symmetry.

Supplementary References

1 Fang, Y. Q. *et al.* Discovery of Superconductivity in 2M WS2 with Possible Topological Surface States. *Advanced Materials* **31**, doi:10.1002/adma.201901942 (2019).

2 Yuan, Y. H. *et al.* Evidence of anisotropic Majorana bound states in 2M-WS2. *Nature Physics* **15**, 1046-+, doi:10.1038/s41567-019-0576-7 (2019).

3 Damascelli, A. Probing the electronic structure of complex systems by ARPES. *Physica Scripta* **T109**, 61-74, doi:10.1238/Physica.Topical.109a00061 (2004).

4 Norman, M. R., et al. Phenomenology of the low-energy spectral function in high-T c superconductors. *Physical Review B* 57.18 (1998).

5 Lee, W. S. *et al.* Abrupt onset of a second energy gap at the superconducting transition of underdoped Bi2212. *Nature* **450**, 81-84, doi:10.1038/nature06219 (2007).
